# Reactive Oxygen Species Prevent Imiquimod-Induced Psoriatic Dermatitis through Enhancing Regulatory T Cell Function

**DOI:** 10.1371/journal.pone.0091146

**Published:** 2014-03-07

**Authors:** Hyung-Ran Kim, Anbok Lee, Eun-Jeong Choi, Min-Pyo Hong, Jeong-Hae Kie, Woosung Lim, Hyeon Kook Lee, Byung-In Moon, Ju-Young Seoh

**Affiliations:** 1 Department of Microbiology, Ewha Womans University Graduate School of Medicine, Seoul, Korea; 2 Department of Surgery, Ewha Womans University Graduate School of Medicine, Seoul, Korea; 3 College of Arts and Sciences, Boston College, Chestnut Hill, Massachusetts, United States of America; 4 Pathology, National Health Insurance Cooperation Ilsan Hospital, Koyang, Korea; New York University, United States of America

## Abstract

Psoriasis is a chronic inflammatory skin disease resulting from immune dysregulation. Regulatory T cells (Tregs) are important in the prevention of psoriasis. Traditionally, reactive oxygen species (ROS) are known to be implicated in the progression of inflammatory diseases, including psoriasis, but many recent studies suggested the protective role of ROS in immune-mediated diseases. In particular, severe cases of psoriasis vulgaris have been reported to be successfully treated by hyperbaric oxygen therapy (HBOT), which raises tissue level of ROS. Also it was reported that Treg function was closely associated with ROS level. However, it has been only investigated in lowered levels of ROS so far. Thus, in this study, to clarify the relationship between ROS level and Treg function, as well as their role in the pathogenesis of psoriasis, we investigated imiquimod-induced psoriatic dermatitis (PD) in association with Treg function both in elevated and lowered levels of ROS by using knockout mice, such as glutathione peroxidase-1^−/−^ and neutrophil cytosolic factor-1^−/−^ mice, as well as by using HBOT or chemicals, such as 2,3-dimethoxy-1,4-naphthoquinone and N-acetylcysteine. The results consistently showed Tregs were hyperfunctional in elevated levels of ROS, whereas hypofunctional in lowered levels of ROS. In addition, imiquimod-induced PD was attenuated in elevated levels of ROS, whereas aggravated in lowered levels of ROS. For the molecular mechanism that may link ROS level and Treg function, we investigated the expression of an immunoregulatory enzyme, indoleamine 2,3-dioxygenase (IDO) which is induced by ROS, in PD lesions. Taken together, it was implied that appropriately elevated levels of ROS might prevent psoriasis through enhancing IDO expression and Treg function.

## Introduction

Psoriasis is a chronic, remitting and relapsing, inflammatory skin disease that is often associated with systemic manifestation, especially arthritis [1]. Currently, psoriasis is managed by topical treatment with steroids, immunosuppressants and several other agents. Psoralen and ultraviolet A (PUVA) photochemotherapy and other systemic therapies, such as using methotrexate, cyclosporine, oral retinoids and biological therapies, are also in practice. However, many psoriasis patients are still sufferring from frequent relapse, adverse drug effects, and other untoward reactions such as development of non-melanoma skin cancer [2]. Thus, it is still desirable to develop another therapeutic strategy that is more effective and does not induce side effects.

Psoriasis is known to develop as a result of immune dysregulation, in particular hyperfunction of T helper 17 (Th17) cells [3], [4]. In steady state, immune homeostasis is maintained by regulatory T cells (Tregs) that suppress immune effectors including Th17 cells. It was also reported that psoriasis is associated with impaired suppressive function of Tregs [5], [6]. Therefore, in order to restore the dysregulated immune status in psoriasis, it is necessary to suppress immune effectors including Th17 cells and/or to enhance Tregs.

Traditionally, reactive oxygen species (ROS) are known to be implicated in the progression of many inflammatory diseases [7]–[9]. As ROS are highly reactive and interact with many bio-molecules, they are likely to destroy biological structures, promoting cellular damage and tissue destruction. In contrast, many recent evidences are accumulating on the protective role of ROS in immune-mediated diseases. Autoimmune arthritis was aggravated in rodents with lower levels of ROS than wildtype (WT) mice due to defects in ROS-producing enzyme system, such as mutation in the neutrophil cytosolic factor (Ncf)-1 or NADPH oxidase (NOX)2 [10]–[12]. In human, too, many autoimmune diseases develop more frequently in chronic granulomatous disease (CGD) patients with lower level of ROS than normal persons due to defect in ROS-producing NOX [13]. To the contrary, experimentally induced asthmatic inflammation was attenuated in mice with higher level of ROS than WT mice due to the defect of a ROS metabolizing enzyme, glutathione peroxidase-1 (GPx-1) [14]. Atherosclerotic lesions induced by high-fat diet were also decreased in GPx-1^−/−^ mice [15]. In addition, experimental colitis was attenuated in mice with a higher level of ROS due to defect in a non-enzymatic anti-oxidant, peroxiredoxin II [16].

These clinical and experimental observations implicated the immunoregulatory role of ROS [17]. In particular, Treg function seems to be closely linked to ROS level. Tregs isolated from mice with a lower level of ROS (Ncf1^−/−^) was hypofunctional, comparing to WT Tregs [18]. Tregs were also hypofunctional in lowered levels of ROS prepared in vitro by addition of anti-oxidants or NOX inhibitors [18]. Consequently, Treg function seems to be closely associated with ROS level, but so far it has only been investigated in lowered levels of ROS. Therefore, in the present study, we investigated the suppressive function of Tregs isolated from mice with a higher level (GPx-1^−/−^), as well as from mice with a lower level (Ncf1^−/−^) of ROS than WT mice, in order to clarify the relationship between ROS level and Treg function. We also investigated Treg function in elevated and lowered levels of ROS prepared in vitro by using chemicals that increase or decrease ROS. In addition, we investigated the susceptibility of GPx-1^−/−^ and Ncf1^−/−^ mice to an experimentally-induced psoriatic dermatitis (PD). Next, for the purpose of immunomodulation, we investigated the susceptibility of WT mice with elevated and lowered levels of ROS prepared by tissue hyperoxygenation or administration of an anti-oxidant to the murine models of PD.

Hyperbaric oxygen therapy (HBOT) is defined as breathing pure (100%) oxygen at increased atmospheric pressure. HBOT is a standard therapy for decompression sickness, gas embolism and CO poisoning [19], [20]. HBOT is also effective for gas gangrene, anaerobic infection, diabetic foot, Buerger’s disease and other oxygen-deficient conditions [21], [22]. In addition, HBOT has been proved effective in the healing of chronic wound, such as radiation-induced soft tissue necrosis [23]–[26]. Meanwhile, many studies reported the therapeutic or preventive effect of HBOT in various kinds of inflammatory or immune-mediated diseases, such as systemic lupus erythematosus [27], [28], atherosclerosis [29], collagen-induced arthritis [30], Crohn’s disease [31], ulcerative colitis [32], [33] and atopic dermatitis [34], although these diseases are not included in the current indication of HBOT. In particular, Butler et al. (2009) reported successful treatment of two severe cases of psoriasis vulgaris by HBOT [35]. Inflammatory and anti-inflammatory cytokines have been investigated in several studies [29], but the therapeutic mechanism of HBOT in immune-mediated diseases still remains enigmatic. Recently, Faleo et al (2012) reported that HBOT prevented autoimmune diabetes in NOD mice [36]. They showed for the first time that the frequency of FoxP3^+^ Tregs was increased in the spleen and pancreatic islets of the mice treated with HBOT. In consequence, it was suggested that Tregs may play an important role in the immunoregulatory mechanism of HBOT. It is well-known that HBOT increases cellular level of ROS according to tissue hyperoxia [37], [38]. Therefore, it can be hypothesized that HBOT may modulate immune-mediated diseases by enhancing Treg function.

For the molecular mechanism of immunomodulation by ROS, we investigated tissue expression of indoleamine 2,3-dioxygenase (IDO). IDO is an immunoregulatory enzyme that can be induced by ROS [39]–[41]. Moreover, the enzyme activity of IDO can be enhanced in high levels of ROS, as superoxide radical acts as a cofactor of IDO [42]. IDO catabolizes the essential amino acid tryptophan into the stable metabolite, kynurenine [42]. Consequently, IDO depletes tryptophan from the environment, thus starving effector cells, and results in immune suppression. It was also found that tryptophan depletion resulted in inhibition of Th17 cell differentiation and expansion of Foxp3^+^ Tregs [40], [43], [44]. Therefore, IDO might be a molecular cue that links ROS and Treg, participating in the immunoregulatory mechanism of ROS.

The results consistently showed that Tregs were hyperfunctional and PD was attenuated in elevated levels of ROS, whereas Tregs were hypofunctional and PD was aggravated in lowered levels of ROS. In addition, it was implied that induction of IDO expression by ROS might contribute to the preparation of immunosuppressive environment that prevent inflammatory reaction in the lesions of PD.

## Materials and Methods

### Mice

Ncf1 (p47^phox^)-deficient mouse strain (B6(Cg)-*Ncf1^m1J/^*J) and GPx1^−/−^ mice on the 57BL/6 background as well as the C57BL/6 WT mice were housed under specific pathogen-free conditions at Ewha Womans University [14], [18]. This study was performed according to Korean Food and Drug Administration guidelines and was specifically approved by the Institutional Animal Care and Use Committee of Ewha Womans University Graduate School of Medicine (Permit Number: 10-0133).

### Measurement of Intracellular ROS Level

Spleens were removed from sacrificed mice and single cell suspension was prepared by squeezing on a cell strainer (70 µm, BD Biosciences, San Jose, CA). CD4^+^ cells were isolated using CD4 T Lymphocyte Enrichment Set (BD Biosciences) and were stimulated with plate-coated anti-CD3e (50 ng/well) and soluble anti-CD28 (1 µg/mL, e-Biosciences) in round-bottomed 96-well plates. Twenty four hours later, the cells were harvested and stained with 10 µM 2,7-dichloro-fluorescein diacetate (DC-FDA, Sigma, St. Louis, MO) for 30 min at 37°C. Then the cells were washed with PBS and were immediately measured for DC-FDA fluorescence using FACSCalibur (BD Biosciences).

### Imiquimod-induced PD

Mice at 6 to 8 wk of age received a daily topical dose of 80 mg per mouse of commercially available imiquimod cream (5%) (Aldara; 3M Health Care, Leicestershire, UK) on the shaved back skin for 5 or 6 consecutive days. Control mice were treated similarly with a control vehicle cream (Vaseline Lanette cream; Fagron, St. Paul, MN). An objective scoring system based on the clinical Psoriasis Area and Severity Index (PASI), except for the affected skin area, was applied to score the severity of inflammation of the back skin [45]. Thus, erythema, scaling, and thickening were scored independently on a scale from 0 to 4: 0, none; 1, slight; 2, moderate; 3, marked; 4, very marked. To increase or decrease tissue level of ROS, some WT mice were treated with HBOT or N-acetylcysteine (NAC, Sigma) once a day from three days before application of imiquimod to the day before investigation, except for the first two days of imiquimod application. NAC (40 mM) was orally administered in drinking water.

### HBOT

A hyperbaric oxygen chamber for animal study was purchased from Particla (Daejeon, South Korea). The HBOT protocol is conducted with 100% O^2^ at 2.5 atm for 90 min after 10 min of compression, and then followed by 30 min of decompression.

### Histological Examination

Excised skins and ears were fixed in 4% paraformaldehyde for 16 h and embedded in paraffin. Thin, 5 µm sections were stained with hematoxylin and eosin (H&E). Epidermal thickening, elongation of rete ridges with hyperkeratosis and parakeratosis were observed in association with vascular dilatation and inflammatory cell infiltration in dermis. Epidermal thickness was measured by using image analysis software (Image J 1.47v, NIH, USA).

### Immunohistochemistry

Sections were deparaffinated in xylene, dehydrated in ethanol and washed in PBS followed by successive permeabilization steps (with Triton 0.2% in PBS). Endogenous peroxidase was blocked with hydrogen peroxide (5% in PBS) for 30 min and the sections were subjected to heat-induced antigen retrieval step before incubation with a universal blocking solution (Dako, Glostrup, Denmark) for 30 min. Then, the sections were incubated with anti-IDO (rabbit polyclonal antibody, Abcam, Cambridge, UK) and Universal LSAB™+ Kit/HRP, Rabbit/Mouse/Goat (Dako), followed by developing using DAB as substrate.

### Preparation of Cells

Single cell suspension was prepared from the spleen, as previously described. After erythrocytes were lysed with RBC lysis buffer (eBioscience, San Diego, CA), CD4^+^CD25^+^ fraction was separated using a regulatory T cell isolation kit purchased from Miltenyi Biotech (Auburn, CA). CD4^+^CD25^−^ cells were also isolated and used for effector T cells (Teffs). For the purity check, the cells were stained with anti-CD4-FITC (H129.19) and anti-CD25-PE (PC61) purchased from BD Biosciences. Then, the cells were further stained for intranuclear FoxP3, using mouse regulatory T cell staining kit (eBiosciences, San Diego, CA) and anti-FoxP3-PerCP-Cyanine5.5 (FJK-16s). The stained cells were analyzed using FACSCalibur. Teffs were labeled with carboxyfluorescein diacetate succinimidyl ester (CFSE, Invitrogen, Carlsbad, CA) by incubating in 1 µM CFSE in protein-free PBS for 5 min at 37°C, to trace the proliferative response [46]. For the purification of dendritic cells (DCs), CD11c^+^ cells were isolated using a spleen dissociation medium (StemCell technologies, Vancouver, BC), density gradient centrifugation over 15.5% Accudenz (Accurate Chemical & Scientific, Westbury, NY), and immunomagnetic selection using anti-CD11c-PE (BD Biosciences) and microbead anti-PE (Miltenyi Biotec). The purity of CD11c^+^ cells was consistently over 95%. Sometimes, cells were prepared 24 hours after intraperitoneal injection of NAC (500 mg/kg) or 2,3-dimethoxy-1,4-naphthoquinone (DMNQ, A.G. Scientific, San Diego, CA; 25 mg/kg).

### *In vitro* Suppressive Assay

For the functional assessment of the suppressive activity of Tregs, Teff proliferation was compared in the absence and presence of Tregs. However, a small percentage of CD4^+^CD25^+^ T cells are not Tregs and do not express FoxP3, and the isolated Treg samples were not pure based on FoxP3 expression. In the present study, 86.6 ∼ 91.4% of the isolated Treg fractions were CD4^+^FoxP3^+^ (Figure S1). To ensure optimal reproducibility of the suppression assays, the purity degree of the Treg samples was taken into account when the cells were plated so that the final ratio of CD4^+^FoxP3^+^ Tregs to CD4^+^FoxP3^−^ Teff was as close as possible to 1∶1 [18]. For assessing the proliferative responsiveness, 10^4^ CFSE-labeled CD4^+^FoxP3^−^ Teffs and 2×10^3^ DCs were cultured in DMEM supplemented with 10% FCS (Hyclone, Logan, UT) in round-bottomed 96-well plates by stimulating with 1 µg/mL of soluble anti-CD3e (e-Biosciences). On the 3^rd^ day of culture, the cells were harvested for staining with anti-CD4-PerCP (BD Biosciences). The stained cells were acquired using FACSCalibur to be analyzed by Winlist software (Verity, Topsham, ME). Precursor frequency (Pf) was estimated for the cells exclusively gated for CD4^+^ live cells according to the scattering characteristics, using the proliferation wizard of Modifit software (Verity), as described elsewhere [46], [47]. For analysis of Treg function, 1 or 0.5×10^3^ CD4^+^FoxP3^+^ Tregs were added to the cultures, and the Pf values in the absence and presence of Tregs were compared to give rise to suppression (%). When necessary, DMNQ or NAC was added to the cultures to increase or decrease ROS level, respectively.

### Statistics

Data are expressed as the mean ± SD (*n* = 12). Comparison of data was performed using independent Student’s t test. *P* values less than 0.05 was considered statistically significant.

## Results

### ROS Generation from CD4^+^ Cells was Decreased in Ncf1^−/−^ Mice, Whereas Increased in GPx1^−/−^ Mice

**Figure 1:**
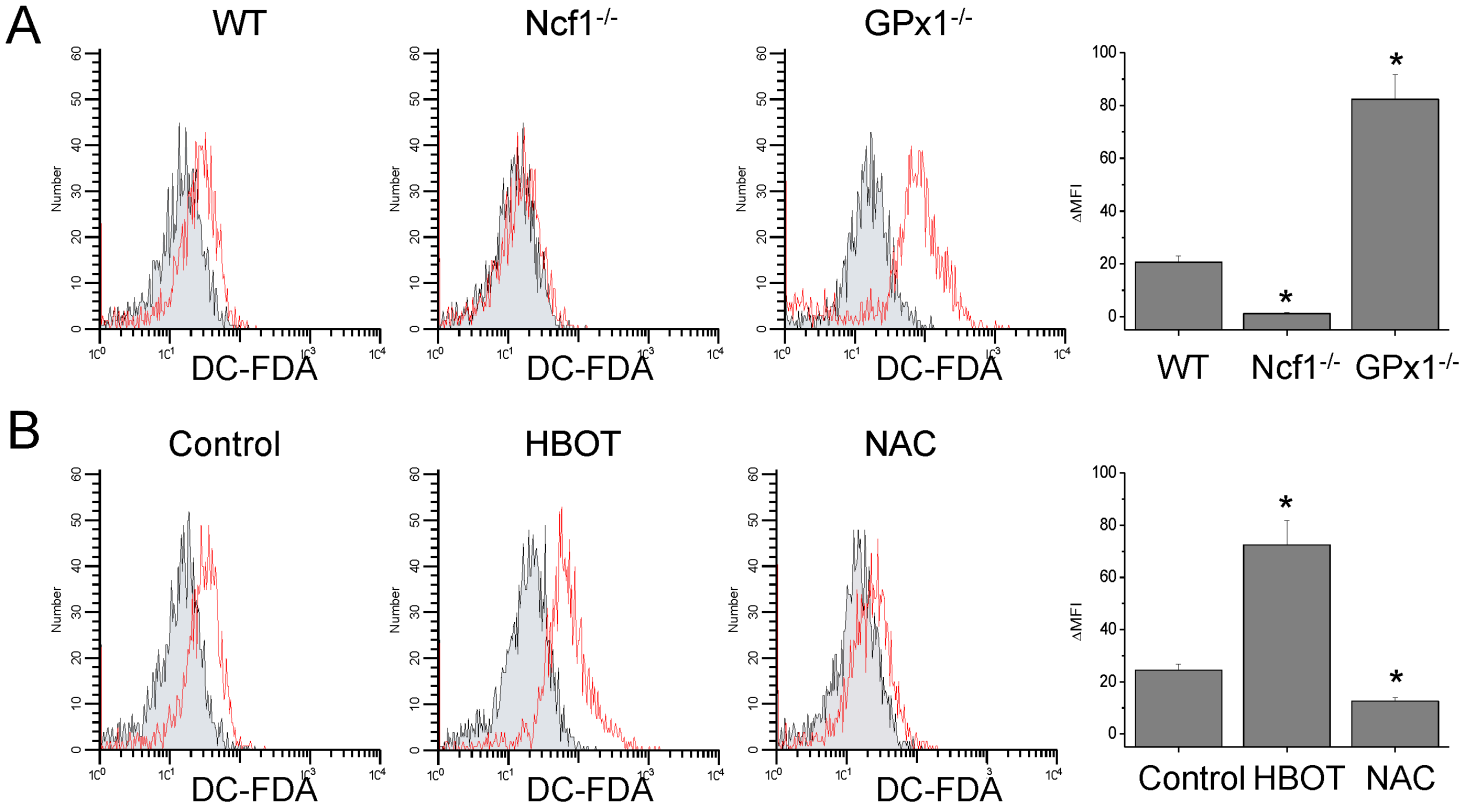
ROS generation from CD4^+^ cells was decreased in Ncf1^−/−^ mice, whereas increased in GPx1^−/−^ mice. (A) Stimulation with anti-CD3e and anti-CD28 induced emission of DC-FDA fluorescence from CD4^+^ cells, reflecting ROS generation. Black lines with shaded area indicate unstained cells, while red lines indicate cells stained with DC-FDA. (B) Treatment of WT mice with HBOT or NAC for 3 days increased or decreased ROS generation from CD4^+^ cells, respectively. ΔMFI indicate the difference of the values of geometric mean fluorescence intensity (MFI) of the stained cells and unstained cells. Data are mean ± SD (n = 12). *P<.05, compared with WT or control.

Stimulation with anti-CD3e and anti-CD28 induced emission of DC-FDA fluorescence from CD4^+^ cells, reflecting ROS generation (Fig. 1). Compared with WT CD4^+^ cells, Ncf1^−/−^ CD4^+^ cells generated less, whereas GPx-1^−/−^ CD4^+^ cells generated more amounts of ROS (Fig. 1A). Treatment of WT mice with HBOT or NAC for 3 days increased or decreased ROS generation from CD4^+^ cells, suggesting elevated or lowered intracellular levels of ROS, respectively (Fig. 1B).

### Imiquimod-induced PD was Aggravated in Ncf1^−/−^ Mice, Whereas Attenuated in GPx-1^−/−^ Mice

**Figure 2:**
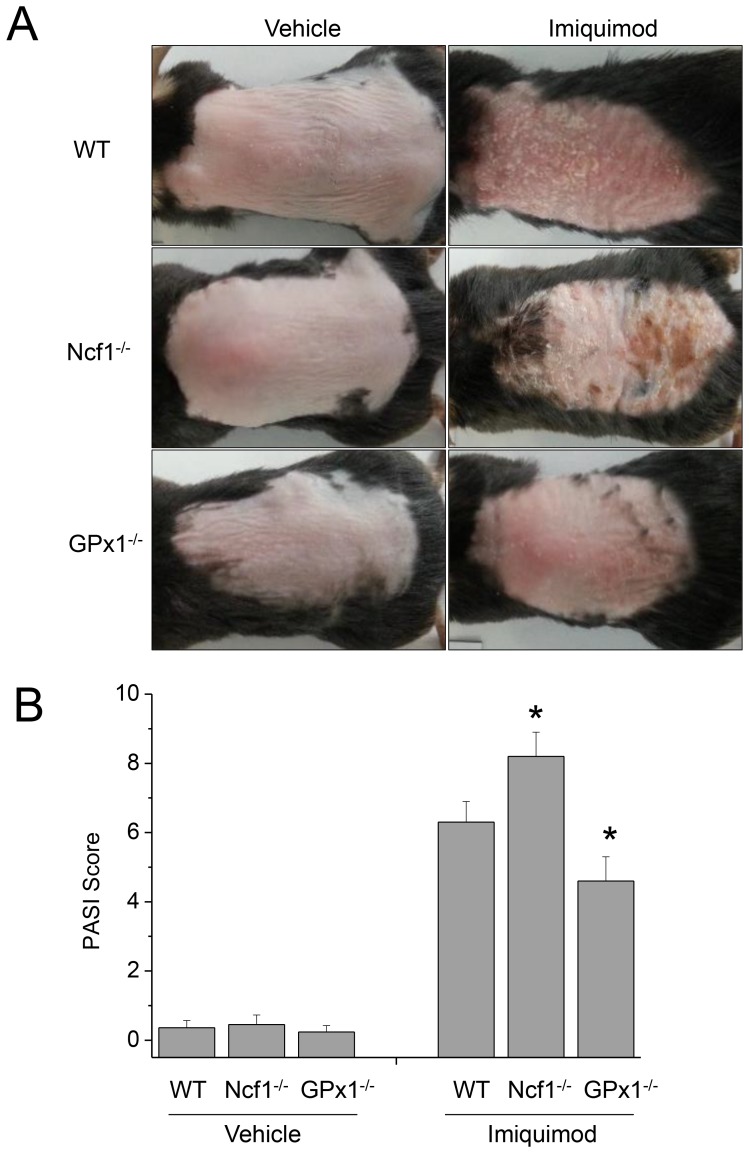
Imiquimod-induced PD was aggravated in Ncf1^−/−^ mice, while attenuated in GPx-1^−/−^ mice. (A) The back skins of the WT, Ncf1^−/−^ and GPx1^−/−^ mice displayed different grades of erythema, scaling and thickening. Photographs were taken on the 7^th^ day after the application of imiquimod for 6 consecutive days. (B) PASI scoring shows more and less severe PD in Ncf1^−/−^ and GPx-1^−/−^ mice, respectively. Data are mean ± SD (n = 12). *P<.05, compared with WT.

**Figure 3:**
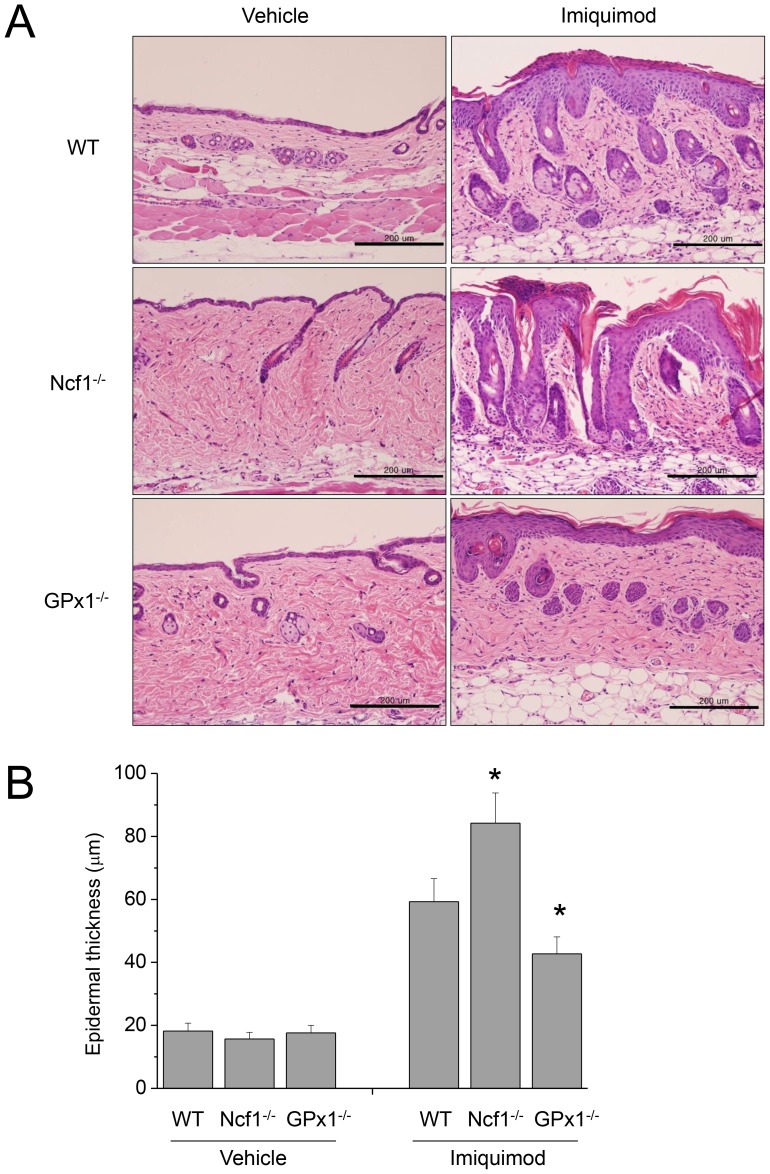
Histological examination of imiquimod-induced PD in Ncf1^−/−^ and GPx-1^−/−^ mice. (A) H&E-stained sections of the PD lesions in WT mice show epidermal thickening and elongation of rete ridges, reflecting the hyperproliferative state, with hyperkeratosis and parakeratosis. The pathologic findings are more prominent in Ncf1^−/−^ mice whereas attenuated in GPx-1^−/−^ mice. (B) Epidermal thickening was more and less prominent in the lesions of Ncf1^−/−^ and GPx-1^−/−^ mice, respectively. Data are mean ± SD (n = 12). *P<.05, compared with WT. Scale bar is 200 µm.

After the application of imiquimod for 6 consecutive days, the back skins of the WT, Ncf1^−/−^ and GPx1^−/−^ mice displayed different grades of erythema, scaling and thickening (Fig. 2). Skin lesions were more and less severe in terms of erythema, scaling and thickness (PASI score) in Ncf1^−/−^ and GPx1^−/−^ mice, respectively. Histological examination of the H&E-stained sections from the PD lesions showed epidermal thickening and elongation of rete ridges, reflecting the hyperproliferative state, with hyperkeratosis and parakeratosis (Fig. 3). The histology of the control skins treated with vehicle was not different between WT, Ncf1^−/−^ and GPx1^−/−^ mice. On the other hand, the histology of the PD lesions was substantially distinguished between WT, Ncf1^−/−^ and GPx1^−/−^ mice; epidermal thickening and elongation of rete ridges were more prominent, hyperkeratosis and parakeratosis were exaggerated and parakeratotic microabscesses were often observed, vascular dilatation and inflammatory cell infiltration were more prominent in dermis of PD in Ncf1^−/−^ mice; whereas all the pathological findings of PD were less prominent in GPx1^−/−^ mice.

### HBOT Attenuated Whereas NAC Aggravated Imiquimod-induced PD

**Figure 4:**
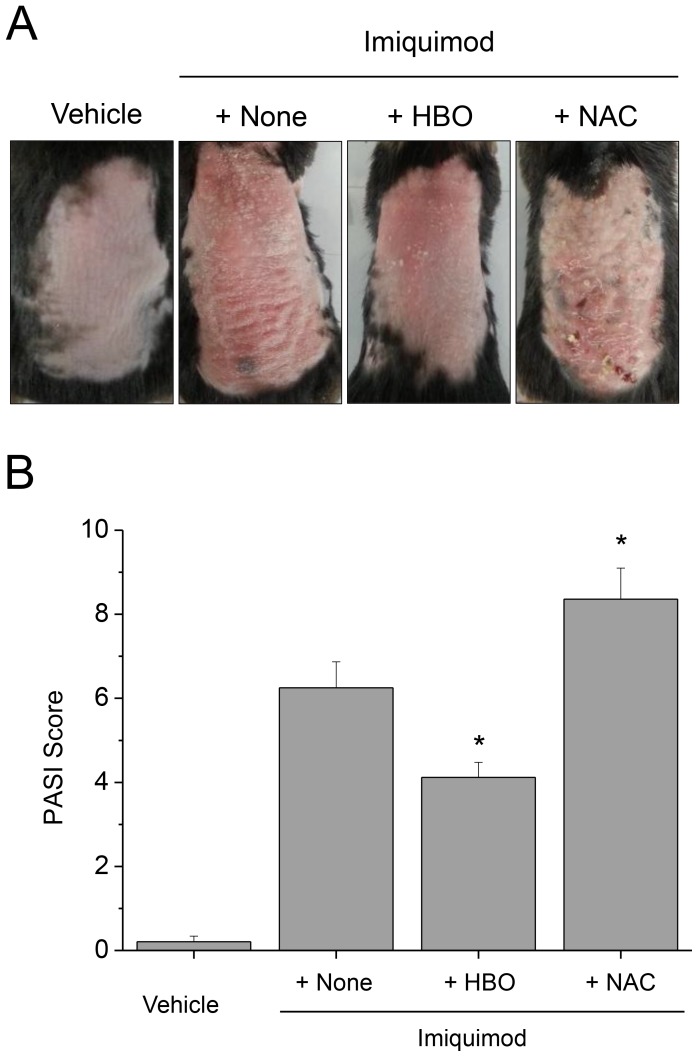
Gross examination show HBOT attenuated, whereas administration of NAC aggravated imiquimod-induced PD. Gross examination show that the PD lesions of the mice treated with HBOT are less severe, whereas those with NAC are more severe, in terms of PASI score. HBO, hyperbaric oxygen; NAC, N-acetylcysteine. Data are mean ± SD (n = 12). *P<.05, compared with the mice treated with imiquimod only.

**Figure 5:**
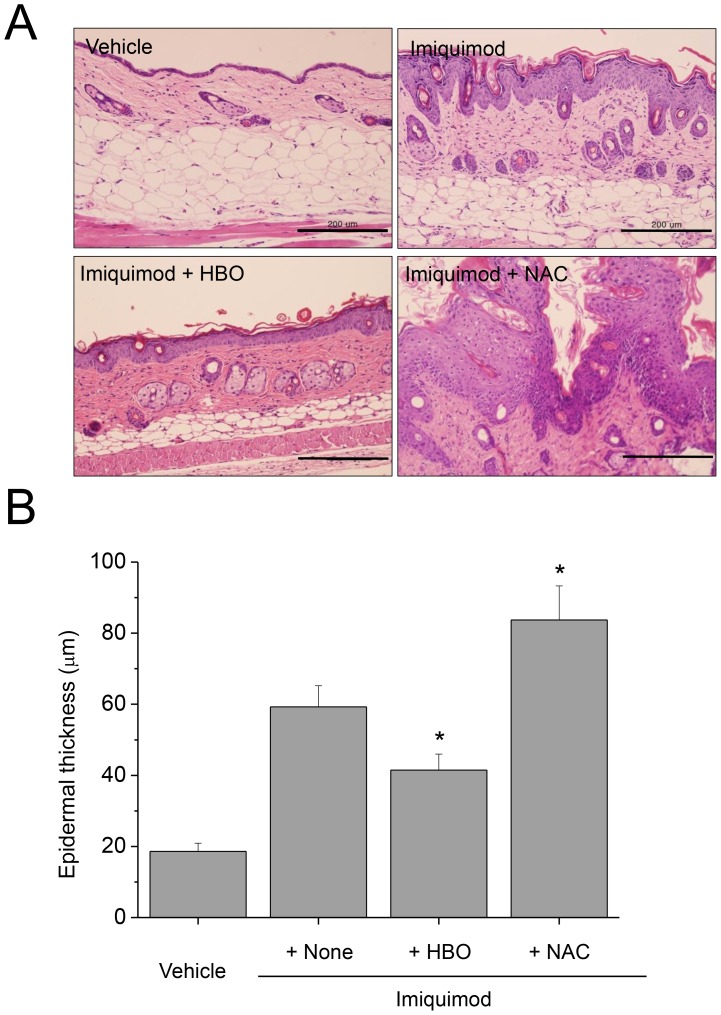
Histologic examination show HBOT attenuated, whereas administration of NAC aggravated imiquimod-induced PD. (A) Pathological findings of PD, as shown in Fig. 2A, are less prominent in the groups treated with HBOT, whereas more prominent in the group treated with NAC. (B) Epidermal thickening was decreased in the groups treated with HBOT, whereas increased in the group treated with NAC. HBO, hyperbaric oxygen; NAC, N-acetylcysteine. Scale bar is 200 µm. Data are mean ± SD (n = 12). *P<.05, compared with the mice treated with imiquimod only.

Gross examination of the PD lesions of the mice treated with HBOT showed less severity, whereas those treated with NAC showed more severity, in terms of PASI score (Fig. 4). Histological examination also showed less and more severe pathological findings of PD in the mice treated with HBOT and those treated with NAC, respectively (Fig. 5).

### HBOT Enhanced Whereas NAC Reduced IDO Expression

**Figure 6:**
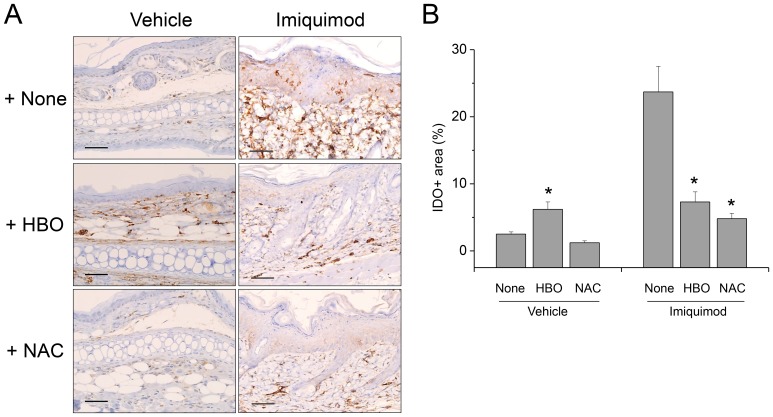
Treatment with HBOT enhanced, whereas administration with NAC reduced IDO expression. (A) In the skins of the control mice treated with vehicle, IDO was very rarely expressed in the dermal layers, but treatment with HBOT increased IDO expression. On the other hand, no noticeable change was observed in the skins of the mice administered with NAC. In the lesions of imiquimod-induced PD, IDO expression was remarkably increased mainly in dermal layer, but was also observed in epidermal layer. Meanwhile, in the lesions of the mice treated with HBOT, IDO expression was not enhanced, compared with the control skins, and substantially weaker, compared with the PD lesions in the mice not treated. Administration with NAC also reduced IDO expression. Scale bar is 100 µm. (B) Image analysis showed a significant increase of the IDO-expressing area in the skins of the control mice treated with HBOT. Meanwhile, in the lesions of PD, IDO-expressing area was decreased by treatment with HBOT or NAC. Data are mean ± SD (n = 12). *P<.05, compared with None.

In the control mouse skins treated with vehicle, IDO was very rarely expressed in the dermal layers, but treatment with HBOT increased IDO expression, suggesting elevated tissue level of ROS (Fig. 6). Most of the IDO-expressing cells were observed in dermal layer. On the other hand, no noticeable change was observed in the skins of the mice administered with NAC. IDO expression was remarkably increased in the lesions of PD mainly in dermal layer but was also observed in epidermal layer. Treatment with HBOT decreased IDO-expressing area in the lesions of PD significantly but it was still strongly expressed in dermal layer. Administration of NAC also reduced IDO expression.

### Suppressive Treg Function was Closely Associated with ROS Level

**Figure 7:**
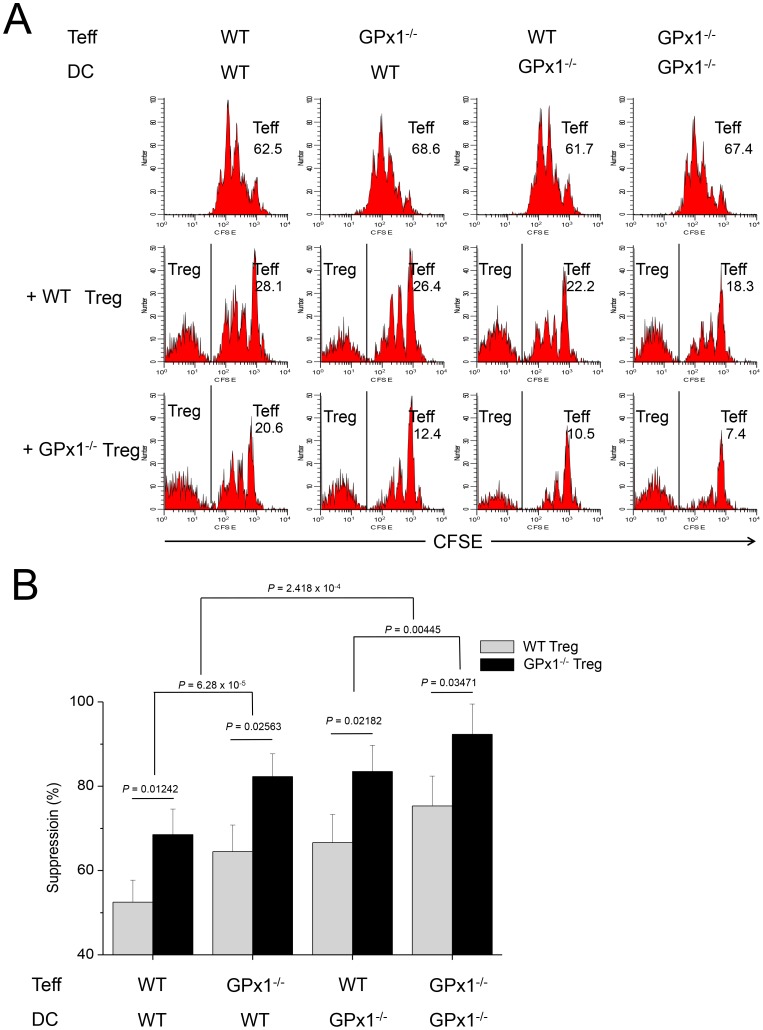
GPx1^−/−^ Tregs were hyperfunctional than WT Tregs. (A) A representative panel of FACS plots for the analysis of suppressive function of Tregs from GPx1^−/−^ mice. On the 3^rd^ day of culture, live CD4^+^ cells were gated for analysis. The first row shows proliferation of Teffs from the WT or GPx1^−/−^ mice in the cross combination with DCs from the WT or GPx1^−/−^ mice, in the absence of Tregs. The 2^nd^ and 3^rd^ rows show proliferation of Teffs in the presence of Tregs from the WT and GPx1^−/−^ mice, respectively. The numbers indicate precursor frequency (%) of Teffs. In the 2^nd^ and 3^rd^ rows, precursor frequency was calculated after CFSE^−^ Tregs were excluded. Teffs were less proliferative in the cocultures with GPx1^−/−^ Tregs than with WT Tregs, suggesting GPx1^−/−^ Tregs were hyperfunctional in the suppression of Teff proliferation, comparing to WT Tregs. (B) Comparison of the suppressive functions of WT and GPx1^−/−^ Tregs in the cocultures with DCs and Teffs from the WT or GPx1^−/−^ mice showed the stronger suppressive power of Tregs as the more types of cells were from GPx1^−/−^ mice, suggesting that Treg function was closely associated with ROS level. Data are mean ± SD (n = 12).

**Figure 8:**
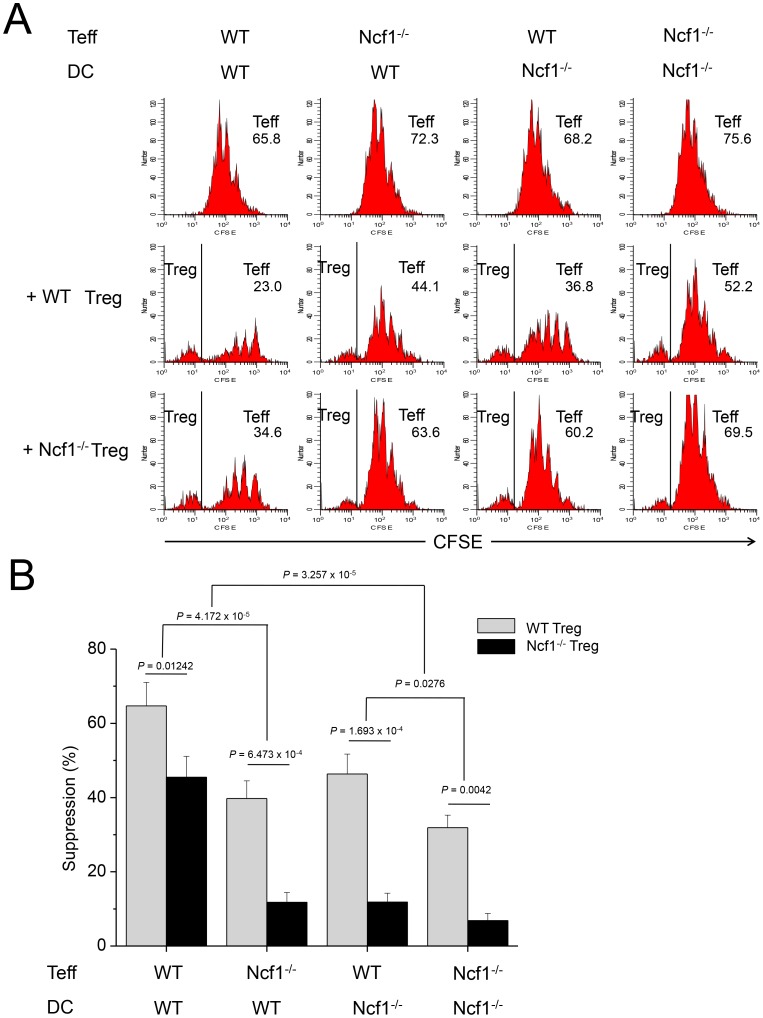
Ncf1^−/−^ Tregs were hypofunctional than WT Tregs. (A) A representative panel of FACS plots for the analysis of suppressive function of Tregs from Ncf1^−/−^ mice. On the 3^rd^ day of culture, live CD4^+^ cells were gated for analysis. The first row shows proliferation of Teffs from the WT or Ncf1^−/−^ mice in the cross combination with DCs from the WT or Ncf1^−/−^ mice, in the absence of Tregs. The 2^nd^ and 3^rd^ rows show proliferation of Teffs in the presence of Tregs from the WT and Ncf1^−/−^ mice, respectively. The numbers indicate precursor frequency (%) of Teffs. In the 2^nd^ and 3^rd^ rows, precursor frequency was calculated after CFSE^−^ Tregs were excluded. Teffs were more proliferative in the cocultures with Ncf1^−/−^ Tregs than with WT Tregs, suggesting Ncf1^−/−^ Tregs were hypofunctional in the suppression of Teff proliferation, comparing to WT Tregs. (B) Comparison of the suppressive functions of WT and Ncf1^−/−^ Tregs in the cocultures with DCs and Teffs from the WT or Ncf1^−/−^ mice showed the weaker suppressive power of Tregs as the more types of cells were from Ncf1^−/−^ mice, suggesting that Treg function was dependent on ROS level. Data are mean ± SD (n = 12).

Teffs in the cultures with GPx1^−/−^ Tregs were less proliferative than those with WT Tregs, suggesting GPx1^−/−^ Tregs were hyper-functional in the suppression of Teff proliferation than WT Tregs (Fig. 7A). A comparison of the suppression by Tregs in the cultures of cross-combination of WT or GPx1^−/−^ Tregs, WT or GPx1^−/−^ Teffs, and WT or GPx1^−/−^ DCs, showed the most powerful suppression in the cultures in which all the 3 kinds of cells, Tregs, Teff and DCs came from GPx1^−/−^ mice (Fig. 7B). The second most powerful suppression was observed in the cultures in which two kinds among the three were from GPx1^−/−^ mice. The next was in the cultures in which any one of the three kinds was from GPx1^−/−^ mice, and the weakest suppression was observed in the cultures in which all the three kinds were from WT mice. As more types of cells were from GPx1^−/−^ mice, the level of ROS in the cultures should be higher, and thus the results suggested Treg function was closely associated with ROS level. Conversely, Teffs in the cultures with Ncf1^−/−^ Tregs were more proliferative than those with WT Tregs, suggesting Ncf1^−/−^ Tregs were hypo-functional in the suppression of Teff proliferation than WT Tregs (Fig. 8A). A comparison of the suppression by Tregs in the cultures of cross-combination of WT or Ncf1^−/−^ Tregs, WT or Ncf1^−/−^ Teffs, and WT or Ncf1^−/−^ DCs, showed that the order of suppression was exactly reverse to that in cultures with cells isolated from GPx1^−/−^ mice (Fig. 8B). As more types of cells were from Ncf1^−/−^ mice, the level of ROS in the cultures should be lower, and thus the results also suggested Treg function was closely associated with ROS level.

### DMNQ Enhanced, Whereas NAC Reduced, Treg Function

**Figure 9:**
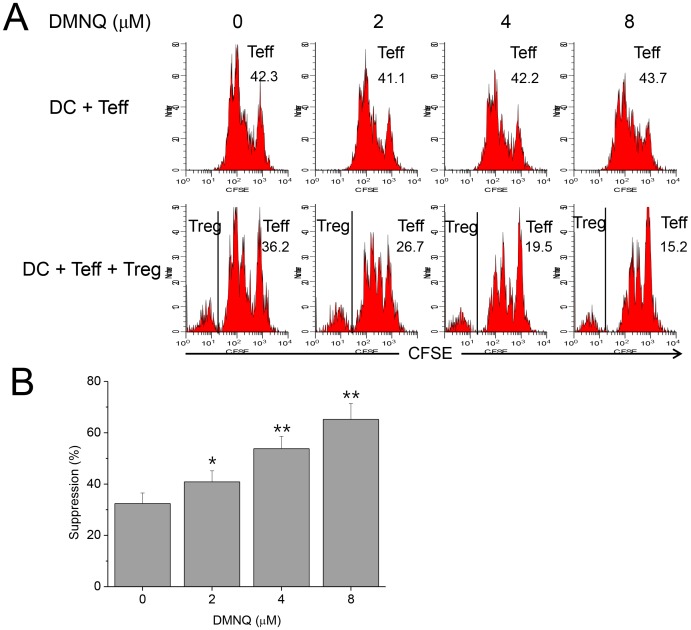
DMNQ enhanced suppressive Treg function. (A) A representative panel of FACS plots for the analysis of suppressive function of Tregs in the presence of various concentrations of DMNQ. DCs, Teffs and Tregs were isolated from WT mice. On the 3^rd^ day of culture, live CD4^+^ cells were gated for analysis. The 1^st^ and 2^nd^ rows show Teff proliferation in the absence and presence of Tregs, respectively. The numbers indicate precursor frequency (%) of Teffs. In the 2^nd^ row, precursor frequency was calculated after CFSE^−^ Tregs were excluded. Teff proliferation in the presence of Tregs decreased as the concentration of DMNQ increased, suggesting DMNQ enhanced Treg function in a dose-dependent manner. (B) Addition of DMNQ in vitro resulted in enhancement of Treg function in a dose-dependent manner. Data are mean ± SD (n = 12). *P<.05 and **P<.01, compared with the control.

**Figure 10:**
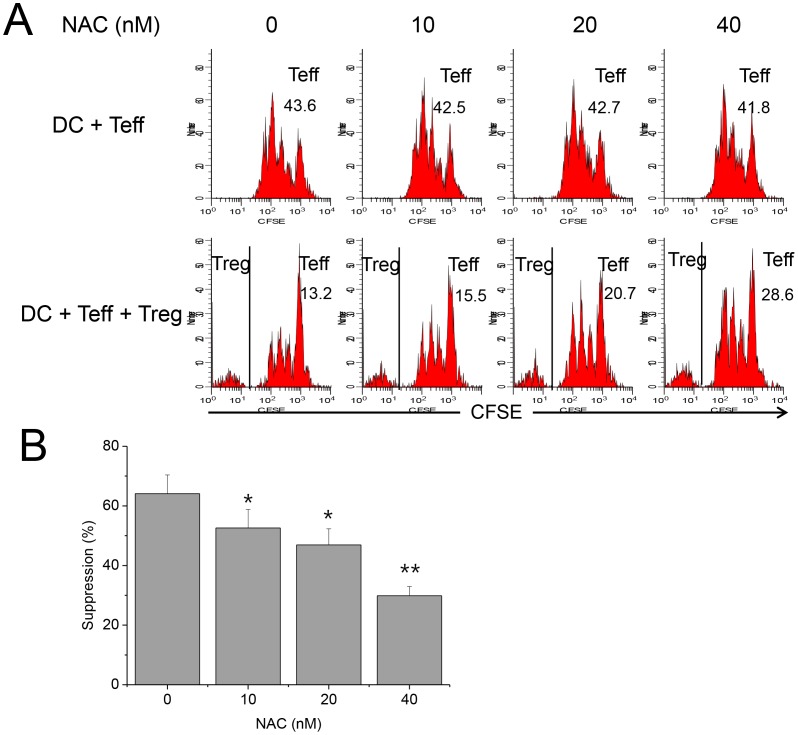
NAC reduced suppressive Treg function. (A) A representative panel of FACS plots for the analysis of suppressive function of Tregs in the presence of various concentrations of NAC. DCs, Teffs and Tregs were isolated from WT mice. On the 3^rd^ day of culture, live CD4^+^ cells were gated for analysis. The 1^st^ and 2^nd^ rows show Teff proliferation in the absence and presence of Tregs, respectively. The numbers indicate precursor frequency (%) of Teffs. In the 2^nd^ row, precursor frequency was calculated after CFSE^−^ Tregs were excluded. Teff proliferation in the presence of Tregs increased as the concentration of NAC increased, suggesting NAC reduced Treg function in a dose-dependent manner. (B) Addition of NAC in vitro reduced Treg function in a dose-dependent manner. Data are mean ± SD (n = 12). *P<.05 and **P<.01, compared with the control.

**Figure 11:**
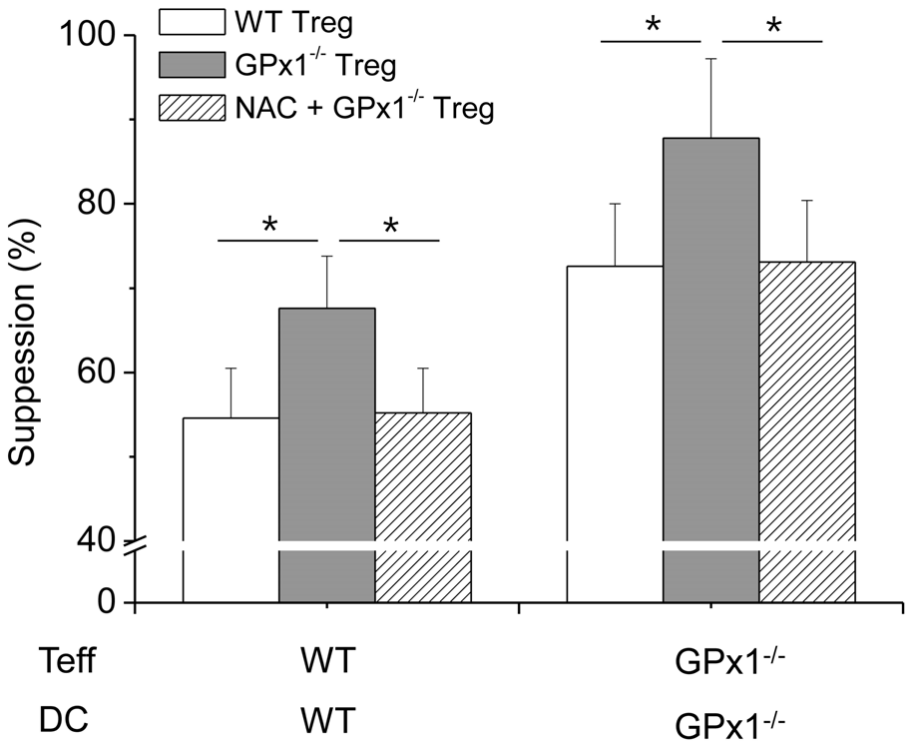
In vivo administration of NAC into GPx1^−/−^ mice reduced Treg function. The suppressive power of Tregs isolated from GPx1^−/−^ mice 24 hours after intraperitoneal injection of NAC was reduced to the level comparable to WT Tregs. Data are mean ± SD (n = 12). *P<.05.

DMNQ induces generation of intracellular ROS, thus elevates ROS level [48]. Tregs were hyperfunctional in the cultures in which DMNQ was added, in a dose-dependent manner, suggesting that Treg function was closely associated with ROS level (Fig. 9). NAC is a reducing agent that decreases ROS level [49]. Tregs were hypofunctional in the cultures in which NAC was added, in a dose-dependent manner, also suggesting that Treg function was closely associated with ROS level (Fig. 10). This result was comparable to that previously reported by Efimova et al., which suggested that NAC, 2-mercaptoethanol, and other NOX inhibitors reduced Treg function [18]. In vivo administration of NAC into GPx1^−/−^ mice reduced Treg function to the level comparable to WT Tregs (Fig. 11). In vivo administration of NAC into WT mice also reduced the suppressive function of Tregs (data not shown). To the contrary, in vivo administration of DMNQ into Ncf1^−/−^ and WT mice enhanced Treg function (data not shown). Taken together, ROS level seemed to be critical in the regulation of Treg function.

## Discussion

In the present study, we demonstrated that imiquimod-induced PD was attenuated in elevated levels of ROS, whereas aggravated in lowered levels of ROS. This observation provides experimental evidence supporting the immunoregulatory role of ROS, that is contradictory to the traditional concept. Traditionally, ROS is implicated in the progression of inflammatory diseases by promoting cellular damage and tissue destruction as well as ageing. At the moment, it is necessary to establish a new conceptual framework where the recent observations and the traditional concept can be compromised.

**Figure 12:**
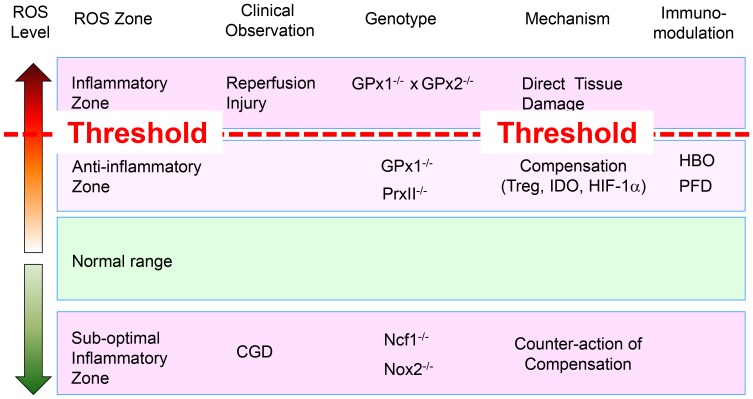
Hypothesis: Immunomodulation depending on ROS level.

We drew a clue from the comparison of GPx-1^−/−^ mice and GPx-1^−/−^×GPx-2^−/−^ mice. According to the traditional concept, ROS accelerates ageing, and lifespan should be shortened in high levels of ROS [50], [51]. However, the life span is not shortened and inflammatory diseases are attenuated in GPx-1^−/−^ mice, although cellular DNA damages are accumulated [52], [53]. In contrast, lifespan is shortened and inflammatory diseases were aggravated in GPx-1^−/−^×GPx-2^−/−^ mice [54]. Thus, we guess that ROS level was elevated but within the tolerable range in GPx-1^−/−^ mice, and too high beyond the tolerable range in GPx-1^−/−^×GPx-2^−/−^ mice. Taken together, we can imagine a threshold level of ROS that divides the moderately high tolerable range and the intolerably higher levels (Fig. 12). In the higher levels such as GPx-1^−/−^×GPx-2^−/−^ mice, inflammatory reactions are augmented due to the direct tissue damage by ROS. Many evident previous observations that contribute to establish the traditional concept of ROS, such as vascular reperfusion injury and other in vitro observations, might fall in this range of intolerably higher levels of ROS [55], [56]. In contrast, as demonstrated previously by others and by us in the present study, inflammatory diseases are attenuated in the moderately high tolerably ranges such as in GPx-1^−/−^ or PrxII^−/−^ mice [14]–[16]. Thus, we suppose some kinds of anti-inflammatory mechanisms are operating in the moderately high tolerable range of ROS. As ROS can induce direct tissue damages at high levels, it would be natural to develop defensive or compensatory mechanisms counteracting the destructive effects of ROS in the body.

In this aspect, enhancement of Treg function depending on ROS level is quite pertinent to counteract the destructive damages induced by ROS, as Tregs suppress every arm of immune response, including Th1, Th2, Th17, B, NK cells and DCs [57], [58]. Hypofunctional Tregs in lowered level of ROS has been reported previously by others as well as by us in the present study [18]. In addition, for the first time in the present study, we demonstrated that Tregs were hyperfunctional in elevated levels of ROS. Thus, it can be argued that Treg function is closely associated with ROS level, and Tregs may contribute to the defense against tissue damages induced by ROS.

Tregs play a critical role in the prevention of autoimmune diseases [59], and functional impairment of Tregs is important in the pathogenesis of psoriasis [4], [6]. Therefore, restoration or strengthening of impaired Treg function would be a desirable therapeutic strategy for psoriasis. The results of the present study suggested appropriately elevated levels of ROS could enhance Treg function, and thus might attenuate psoriasis. HBOT is an easy and safe way to increase tissue level of ROS, which has been applied for a long time for many clinical situations [60]–[62]. Actually in the present study, we demonstrated that HBOT attenuated a murine model of PD. Successful treatment of human cases of psoriasis vulgaris by HBOT has been also reported [35]. Photo(chemo)therapy has been used for a long time for the treatment of psoriasis, but the exact mechanism of action has not yet been clear. Recently, several studies reported that impaired Treg function in psoriatic patients was restored by phototherapy [63]–[65]. It is well-known that ROS is generated by phototherapy [66], and thus elevation of tissue level of ROS might be an underlying mechanism of phototherapy, which enhances Treg function.

Another molecular mechanism investigated in the present study for the defense against ROS-induced tissue damage is IDO expression. IDO is primarily expressed in antigen-presenting cells, such as dendritic cells and macrophages. As an oxygenase, IDO can be induced by ROS [39]–[41]. In the present study, IDO expression was only rarely expressed in the control skins, but substantially enhanced by HBOT, suggesting that ROS level was increased (Fig. 6). In elevated levels of ROS, the enzyme activity of IDO should be enhanced as superoxide radical acts as a cofactor of IDO [42]. Therefore, enhanced expression and activity of IDO from the beginning through hyperoxygenation by HBOT might contribute to the preparation of immunosuppressive environment preventing inflammatory damages in the PD [67], [68]. On the other hand, IDO pathway is induced in many tissues during inflammation because IDO gene expression is induced by interferons [40], [41], [69]. In the present study, IDO expression was remarkably enhanced in the lesions of PD, compared with the control skins (Fig. 6). Accordingly, IDO expression should be enhanced during or after the inflammatory reactions in the lesions of PD, and might contribute to the feedback regulation. In contrast, in the groups treated with HBOT, IDO expression was not significantly different before and after the development of PD, suggesting that IDO expression was not increased as a consequence of inflammatory reactions. Instead, early expression of IDO from the beginning might contribute to the prevention of PD. On the other hand, in the groups treated with NAC, IDO expression was enhanced after the development of PD, but at a far lower level than that not treated. Thus, it was suggested that treatment with NAC inhibited the expression of IDO in the lesions of PD although inflammatory reaction was developed. Consequently, NAC might inhibit feedback regulation of the inflammatory reaction by IDO, and thus aggravating the inflammatory tissue damages.

Based on the observations made in the present study, we propose that tissue hyperoxygenation by HBOT might be an alternative therapeutic strategy for psoriasis. The current protocol of HBOT within 2 hours at maximum 3 atm induces only reversible biochemical changes [70]–[72]. HBOT is quite safe, as major complications are very rare without sequeale, and there is no noticeable complication such as telangiectasia or skin cancer [60]–[62]. However, for practical application, several important points must be considered. Firstly, at the moment, we cannot specify quantitatively the threshold level of ROS between the higher levels that induce direct tissue damage and the moderately high tolerable range in which anti-inflammatory defensive mechanisms are operating. Secondly, there may be variation in ROS level between individuals and/or along the progression of disease state. These issues must be intensively investigated in the following studies.

In the present study, we demonstrated that ROS level was critical both in the pathogenesis of PD and in the regulation of Treg function. We also demonstrated that tissue hyperoxygenation through HBOT attenuated a murine model of PD. In conclusion, we propose that HBOT might attenuate psoriasis through enhancing Treg function.

## Supporting Information

Figure S1 **Purity of isolated CD4^+^CD25^+^ fraction.** The isolated CD4^+^CD25^+^ fraction is not pure Treg population, in terms of FoxP3 expression. CD4^+^FoxP3^+^ cells ranged from 86.6 ∼ 91.4% (88.2±3.4%, n = 12) in the CD4^+^CD25^+^ fraction.
(TIF)
