# Supplementary figures and images for: Reactive Oxygen Species Prevent Imiquimod-Induced Psoriatic Dermatitis through Enhancing Regulatory T Cell Function
Source: PLoS One. 2014 Mar 7;9(3):e91146. doi: 10.1371/journal.pone.0091146 (PMC3946742; doi:10.1371/journal.pone.0091146)

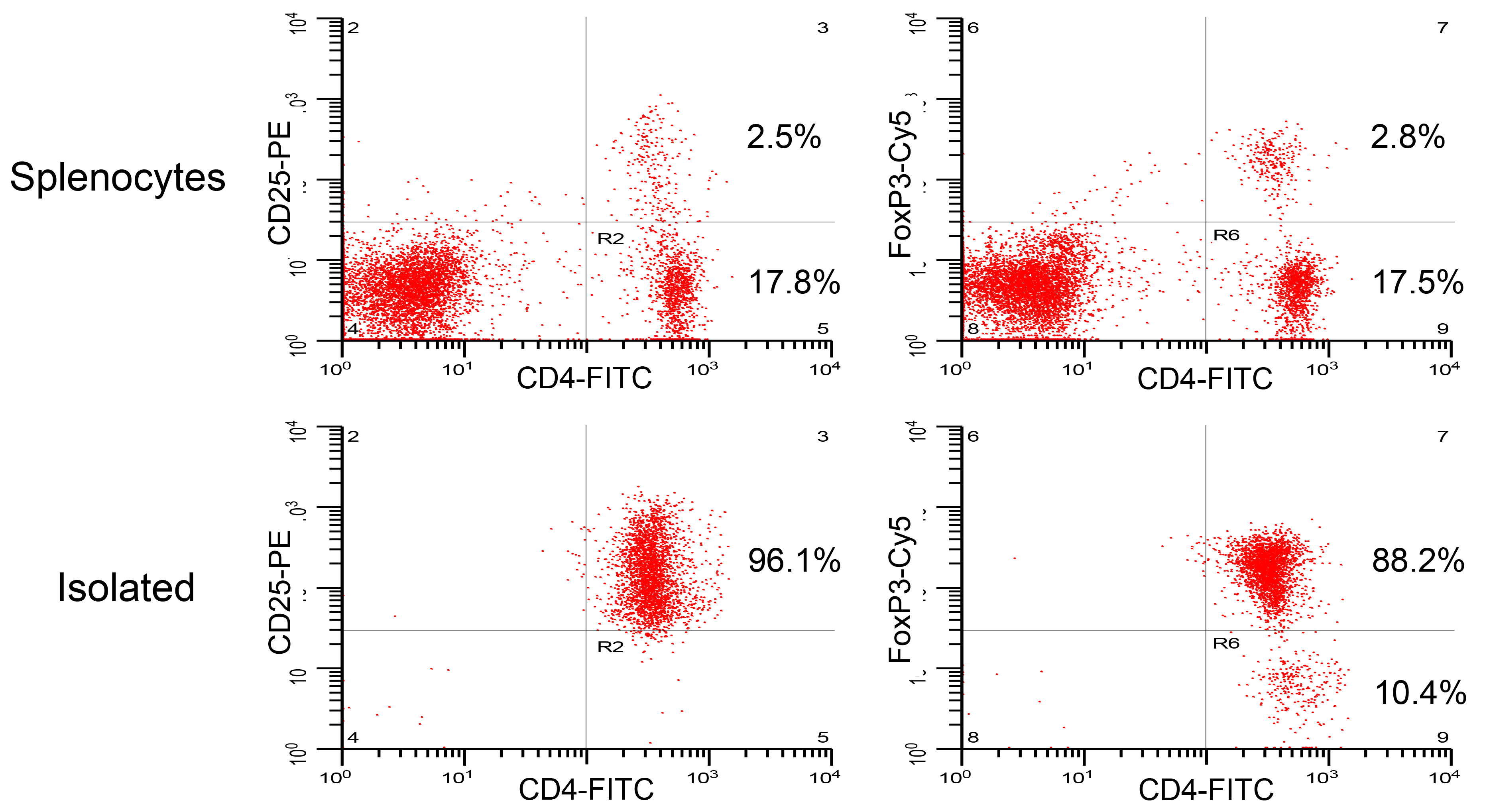

Supplement: Figure S1 — Purity of isolated CD4+CD25+ fraction. The isolated CD4+CD25+ fraction is not pure Treg population, in terms of FoxP3 expression. CD4+FoxP3+ cells ranged from 86.6 ∼ 91.4% (88.2±3.4%, n = 12) in the CD4+CD25+ fraction. (TIF) [file pone.0091146.s001.tif]
